# Annotation of gene product function from high-throughput studies using the Gene Ontology

**DOI:** 10.1093/database/baz007

**Published:** 2019-02-01

**Authors:** Helen Attrill, Pascale Gaudet, Rachael P Huntley, Ruth C Lovering, Stacia R Engel, Sylvain Poux, Kimberly M Van Auken, George Georghiou, Marcus C Chibucos, Tanya Z Berardini, Valerie Wood, Harold Drabkin, Petra Fey, Penelope Garmiri, Midori A Harris, Tony Sawford, Leonore Reiser, Rebecca Tauber, Sabrina Toro, The Gene Ontology Consortium

**Affiliations:** 1 FlyBase, Department of Physiology, Development and Neuroscience, University of Cambridge, Downing Street, Cambridge , UK; 2 CALIPHO group, SIB Swiss Institute of Bioinformatics, Centre Medical Universitaire, rue Michel Servet, CH Geneva, Switzerland; 3 Institute of Cardiovascular Science, University College London, London, UK; 4 *Saccharomyces* Genome Database, Department of Genetics, Stanford University, Porter Drive, Palo Alto, CA, USA; 5 Swiss-Prot group, SIB Swiss Institute of Bioinformatics, Centre Medical Universitaire, rue Michel Servet, CH Geneva, Switzerland; 6 WormBase, Division of Biology and Biological Engineering, California Institute of Technology, E California Blvd, Pasadena, CA, USA; 7 European Molecular Biology Laboratory, European Bioinformatics Institute, Cambridge, UK; 8 Evidence and Conclusion Ontology, University of Maryland School of Medicine, W Baltimore St., Baltimore, MD, USA; 9 The Arabidopsis Information Resource, Phoenix Bioinformatics, Redwood City, CA, USA; 10 PomBase, Cambridge Systems Biology Centre and Department of Biochemistry, University of Cambridge, Sanger Building, Tennis Court Road, Cambridge, UK; 11 Mouse Genome Informatics, Department of Computational Biology and Bioinformatics, The Jackson Laboratory, Main St., Bar Harbor, ME, USA; 12 dictyBase, Biomedical Informatics Center and Center for Genetic Medicine, Northwestern University, Feinberg School of Medicine, North Lake Shore Drive, Chicago, IL, USA; 13 Zebrafish Information Network, University of Oregon, Eugene, OR, USA

## Abstract

High-throughput studies constitute an essential and valued source of information for researchers. However, high-throughput experimental workflows are often complex, with multiple data sets that may contain large numbers of false positives. The representation of high-throughput data in the Gene Ontology (GO) therefore presents a challenging annotation problem, when the overarching goal of GO curation is to provide the most precise view of a gene's role in biology. To address this, representatives from annotation teams within the GO Consortium reviewed high-throughput data annotation practices. We present an annotation framework for high-throughput studies that will facilitate good standards in GO curation and, through the use of new high-throughput evidence codes, increase the visibility of these annotations to the research community.

## Introduction

The Gene Ontology (GO) is one of the most widely used computational resources for assigning functional attributes to genes and their products (1). A standard GO annotation is made by associating a gene product to a GO term supported by an evidence code from the Evidence and Conclusion Ontology (ECO) (2) and the data source for that specific assertion (3). For example, *Caenorhabditis elegans air-2* is annotated to `protein serine/threonine kinase activity’ (GO:0004674), with the evidence code `direct assay evidence used in manual assertion’ (ECO:0000314) linked to the source PMID:15916946. This annotation was based on an *in vitro* kinase assay presented in Han *et al.* (4), demonstrating that AIR-2 can phosphorylate serine 634 in TLK-1. The central role of a GO curator is to interpret the functional data and select terms to best represent a gene's role. Curation using the GO relies on careful and accurate curation to a set of guidelines developed by Consortium participants. Within the GO Consortium (GOC), curators and ontologists meet frequently to ensure that practices are reviewed and kept current (1). GO annotation standards, however, are based on low-throughput experimental set-ups, where the results of experiments can be interpreted in context, accounting for background knowledge about the gene, experimental hypothesis, physiological relevance of the assay and other criteria (5). Curation of high-throughput papers is very different in that it is often not possible to consider the annotation of each gene on a case-by-case basis.

For the purposes of this discussion, it is important to define what characteristics we use to define `low-throughput’ and `high-throughput’ studies. In general, low-throughput studies aim to elucidate the role of a targeted selection of gene products. These studies are usually hypothesis driven, with the experimental design founded on previous knowledge. The workflow tends to be a series of small-scale experiments that either approach the same biological question in multiple ways and/or incrementally extend the characterization to build a more complete biological model. It should first be noted that high-throughput studies encompass a wide variety of experimental methodologies, and those amenable to functional annotation using the GO represent a small subset of such studies. Most high-throughput studies, for example genome-wide association studies and drug screens, fall outside of the remit of the GO curator. Typically, high-throughput experiments apply the same workflow to a large number of genes/gene products often using an automatic or semi-automatic methodology and may provide little or no secondary validation of the results for individual gene products. They address open-ended questions rather than hypothesis-driven questions and the data is usually presented as a data set with the same property assigned to genes/gene products that fall within a given measurement range.

Over the 20 years that GO has been active, there has been a steady increase in the number of publications that contains data generated using high-throughput workflows. With advances in instrumentation and the push to understand complex systems, this growth is set to continue. With the increase in high-throughput data comes the need to usefully disseminate such data to the research community, and to make it FAIR (findable, accessible, interoperable and reusable) (6), such that it can usefully inform ongoing research. For many high-throughput data types, numerous consortia and groups, such as the ProteomeXchange consortium (7), have defined data exchange formats and established standards to describe data. However, for many other high-throughput experiments, data standards do not exist, or, if they do exist, the standards reported often do not include any confidence thresholds, particularly for purely qualitative data sets. The challenge for GO curation is thus to extract useful and accurate annotations from high-throughput data sets that are informative about the physiologically relevant aspects of gene function: biological process, molecular function and cellular component, in a way which minimizes the addition of erroneous annotations.

Despite the increasing number of high-throughput papers, clear guidance on how to curate such data using the GO was lacking. As a result, practices among annotation groups had diverged resulting in inconsistent annotation, inclusion of likely false positives and inconsistent use of evidence codes. Recognizing this, the GOC reviewed its policy to provide a high-throughput annotation framework and a new set of evidence codes that allow clear identification of annotations derived from high-throughput data sets and a set of guidelines to ensure consistent standards of curation for these data. Finally, existing high-throughput annotations were identified and reviewed using the new framework.

## GO annotation of high-throughput data: practices and quality

Consultation with GO annotation groups highlighted differences in how they approached high-throughput data. Some groups only annotated data sets to GO they deemed of high value to their principal research community, others had opted to annotate high-throughput data through different curation streams and others excluded high-throughput papers altogether. For example, the *Saccharomyces* Genome Database (SGD) curated high-throughput data sets for GO using internal guidelines, first developed in 2003 and further refined as large-scale studies, became more common. These annotations were displayed on individual gene pages at SGD separated from the manually curated annotations and clearly marked as having been derived from high-throughput studies. They were made using conventional evidence codes, with an additional `high-throughput’ label in the SGD database.

A review of data in GO showed that the primary problem associated with the use of high-throughput data as a basis to make GO annotations stemmed from the large numbers of erroneous annotations that can be generated from a single study (8). Two main sources of erroneous annotation were identified. First, all high-throughput data sets contain false positives to a degree, which is usually difficult to establish, and, thus curating this data will generate some incorrect annotations. Some high-throughput studies incorporate multiple experimental steps or computational analyses to reduce the level of false positives, but many other studies are not sufficiently rigorous in the identification of false positives to provide the level of certainty required for inclusion in the GO database (see Case study 1). Second, as with low-throughput data, errors may arise from the selection of the incorrect term by the curator, but because of the large number of annotations generated, the impact may be orders of magnitude higher (see Case study 2).

Given the problems with attaching functional attributes to entities from high-throughput studies, the GOC considered whether there should be an embargo on annotating such data using the GO. However, this inflexibility would be a disservice to the curation and research user base. Some high-throughput data sets may provide comprehensive coverage of a particular area of biology not readily tractable by other methods, and, for some genes, such screens provide the only functional data available. Indeed, many annotation groups indicated strongly that they wanted to retain GO annotation of existing high-value data sets, and groups continue to make annotations from new high-throughput papers when they fill a particular knowledge gap. For example, the global analysis of protein localization using green fluorescent protein tagging in fission yeast provides extensive coverage, 4431 proteins, corresponding to ~90% of the proteome (9). This data set was annotated using terms from the GO cellular component aspect by PomBase in 2009 and still includes many proteins that are as yet uncharacterized by low-throughput techniques. Despite containing some false positives, this data set has been of high value to the primary research community, providing an often cited starting point for the subsequent functional analysis of many novel proteins.

Thus, the GOC concluded that although there are examples of high-throughput studies that were not suitable for annotation with GO, other studies can produce high-value annotation, meeting the standards that would allow a curator to assign functional attributes with the degree of confidence required. To facilitate the annotation of high-throughput data sets, GO should provide a mechanism to: (i) clearly distinguish annotations derived from high-throughput workflows and (ii) provide annotation guidelines to help maintain curation accuracy and consistency.

## New high-throughput evidence codes

In high-throughput studies, the same functional property is usually applied to all gene products that fall within a given value bin or classification and therefore assigned GO terms as a set. As annotations from high-throughput papers were previously described using conventional GO evidence codes for experimental data (3), each independent annotation was indistinguishable from those derived from low-throughput studies and the high-throughput provenance was invisible to most users of the GO. Evidence codes are often used as a proxy for confidence, and while it does not follow that a particular annotation derived from an automated computational pipeline is less correct, users tend to give greater weight to experimental evidence. The same is true when comparing high- and low-throughput data, users may use the experimental provenance inform their interpretation.

To allow this discrimination to be made by consumers of GO, five new evidence codes to describe high-throughput experiments have been formulated in concert with the ECO (2). The new codes mirror conventional experimental evidence codes (Table 1) and, in ECO, are children of `high throughput evidence used in manual assertion’ (ECO:0006056; Figure 1), and their low-throughput equivalent, e.g. `high throughput direct assay evidence used in manual assertion’ (ECO:0007005) is a child of `direct assay evidence used in manual assertion’ (ECO:0000314).

**Table 1:** High-throughput evidence codes

ECO ID	Term name	Three-letter GO abbreviation	LTP equivalent ECO ID	LTP equivalent term name	Three-letter GO abbreviation
ECO:0006056	High throughput evidence used in manual assertion	HTP	ECO:0000269	Experimental evidence used in manual assertion	EXP
ECO:0007001	High throughput mutant phenotype evidence used in manual assertion	HMP	ECO:0000315	Mutant phenotype evidence used in manual assertion	IMP
ECO:0007003	High throughput genetic interaction evidence used in manual assertion	HGI	ECO:0000316	Genetic interaction evidence used in manual assertion	IGI
ECO:0007005	High throughput direct assay evidence used in manual assertion	HDA	ECO:0000314	Direct assay evidence used in manual assertion	IDA
ECO:0007007	High throughput expression pattern evidence used in manual assertion	HEP	ECO:0000270	Expression pattern evidence used in manual assertion	IEP

Five new high-throughput evidence codes added to the Evidence and Conclusion Ontology for GO curation are shown in the first two columns (ECO identifier and term name). These are shown next to the equivalent conventional (labeled LTP) term. The standard GO three-letter abbreviation is shown for each code (EXP (Inferred from Experiment), IDA (Inferred from Direct Assay), IMP (Inferred from Mutant Phenotype), IGI (Inferred from Genetic Interaction) and IEP (Inferred from Expression Pattern)). The new high-throughput evidence codes were chosen specifically to mirror those used in conventional GO annotation, so that retrofit of data would be straightforward—many databases use the conventional GO evidence codes and the standard GO annotation exchange file, the gene association file, uses the three-letter GO abbreviation (http://geneontology.org/page/go-annotation-file-formats). Going forward, as groups adopt the gene product association data exchange format for GO annotations, which directly uses ECO codes, there is scope to expand the set of high-throughput evidence codes used by GO curators to capture the specific types of assay used in high-throughput studies, such as immunofluorescence confocal microscopy or functional complementation evidence. Curators that wish to expand the set of high-throughput ECO codes should submit requests directly to ECO http://www.evidenceontology.org/submit_term_request/. Abbreviations: Evidence and Conclusion Ontology identifier (ECO ID), low throughput (LTP).

**Figure 1:**
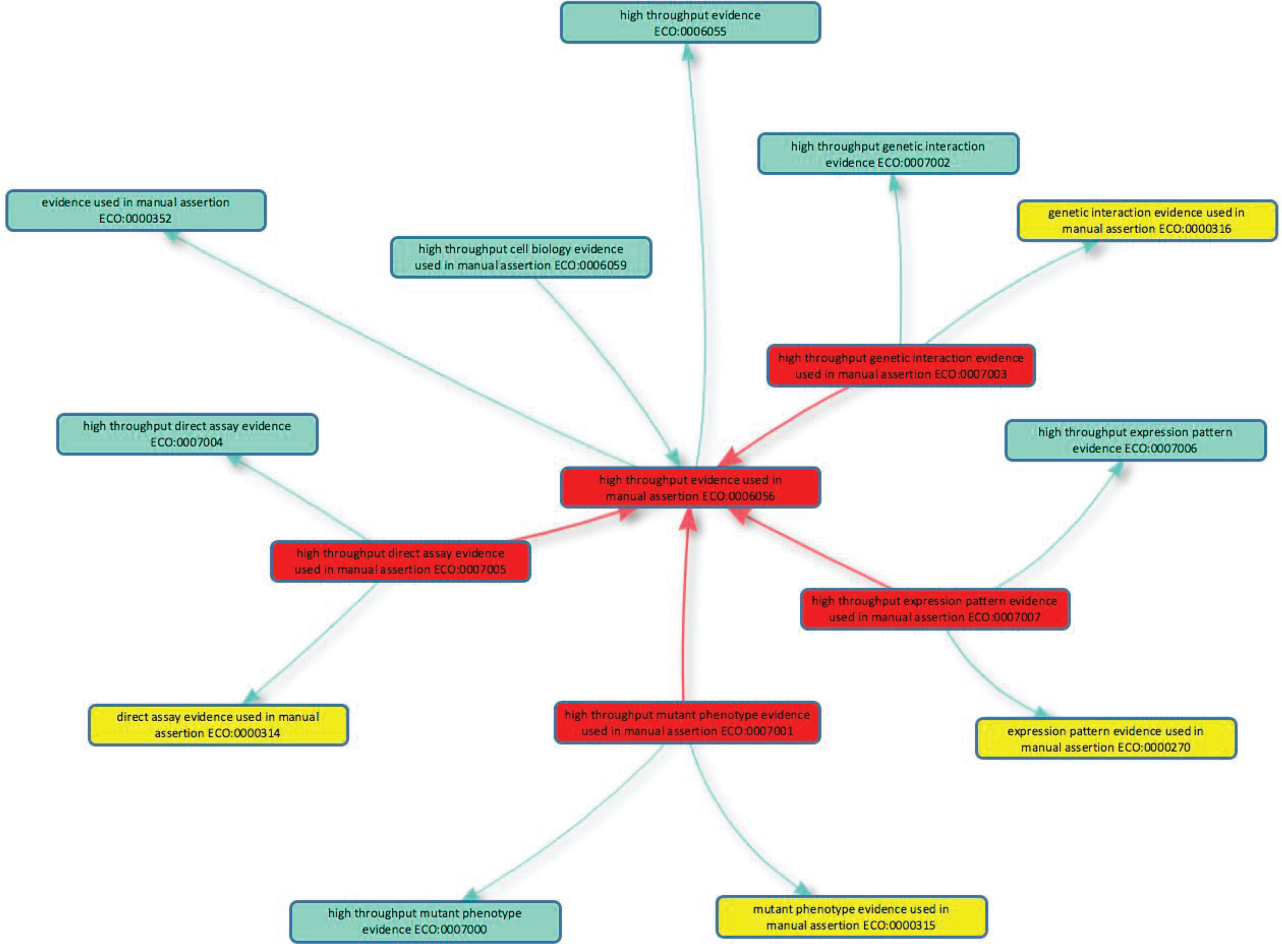
**High-throughput evidence codes to support GO curation.** The five new evidence codes added to the Evidence and Conclusion Ontology for GO curation are shown in red boxes: the parent term `high throughput evidence used in manual assertion’ (ECO:0006056) and four child terms. Is_a relationships with other classes in the Evidence & Conclusion Ontology are shown, including the evidence equivalents used in conventional GO annotation (yellow boxes). The new high-throughput evidence codes should be used by GO curators when annotating high-throughput data in accordance with GOC annotation guidelines. The graph layout was generated using the Ontology Lookup Service (https://www.ebi.ac.uk/ols/index) OLS-graphview.

Clearly marking annotations with high-throughput evidence codes makes the supporting evidence for the annotations more transparent, allows users to weigh them against conflicting or supporting data and to place them within the context of a wider study. Crucially, since the GO is often used to analyze high-throughput data, these data can now be excluded when analyzing or validating data derived from a similar experimental protocol, to avoid reinforcing systematic errors (8). It should be noted that no high-throughput evidence code equivalent to `physical interaction evidence used in manual assertion’ (`IPI’, ECO:0000353) was instantiated as no high-throughput data sets of this type had been used to directly make GO assignments. One reason for this is that GO curators usually look for more supporting evidence that an individual interaction is direct to annotate to protein binding (GO:0005515) or its children. In addition, many databases have their own curation stream for physical interactions and/or these data are captured by specialized molecular interactions resources. Indeed, binary molecular interactions are more comprehensively covered by dedicated curation flows, and curation of high-throughput physical interactions using the GO would lead to duplication of curation effort (10).

## Guidelines to support curation of high-throughput data

The GOC have produced annotation guidelines to accompany the use of high-throughput evidence codes by GO curators. These are available on the GOC wiki [http://wiki.geneontology.org/index.php/Inferred_from_High_Throughput_Experiment_(HTP)]. To ensure consistency across GO, the same basic annotation standards apply to all experimental studies, regardless of throughput, following the existing guidelines developed by the GOC for experimental evidence codes (5) (http://wiki.geneontology.org/index.php/Guide_to_GO_Evidence_Codes#Experimental_Evidence_Codes).

Below are four basic rules for good curation practices of high-throughput data using the GO:

1. Only annotate data with a low false-positive rate: to be suitable for annotation it is especially important that the authors have designed the experiment to minimize the likelihood of false positives. For example, the careful design of experimental set-up, repeated testing, verification by independent screening methods, high-scoring threshold, low false-discovery rate cut-off, identification and exclusion of common contaminants/housekeeping genes and multivariate data analysis (see Case study 3) can all lower the false-positive rate to an acceptable level for annotation.
2. High-throughput data sets do not need to be annotated as a whole set: it should be noted that GO is not a repository for high-throughput data sets—there are more appropriate services dedicated to data housing and displaying which genes/gene products are part of a data set. GO curators can incorporate subsets of annotations derived from high-throughput experiments where they meet the correct criteria, adding high-value biological knowledge of a system. In many instances, data subsets are generated by extra screening steps. Curators may also use statistical cut-offs to select a subset for annotation.A major part of GO curation is the review of existing annotations to ensure that they most accurately represent current biological knowledge. As with conventional annotations, it is expected that annotations evidenced with a high-throughput evidence code can be removed if shown to be incorrect, for example, in the light of new information. The addition of high-throughput evidence codes makes it easier to identify if erroneous annotations come from a high-throughput data set and, in some cases, spotting an obvious outlier should trigger a review of the whole set. Importantly, individual entries from a high-throughput data set can be deleted from the GO annotated data set over time if they are known to be false positives, as part of the ongoing quality assurance process (1).
3. Choose the term with care: as with conventional annotation, curators must assess whether they can confidently assign a GO term based on the experimental output. The term chosen should be directly applicable to the biological question. This is particularly important when dealing with phenotype data—many observed phenotypes are not necessarily indicative of direct involvement in the specific biological processes affected (see Case study 2). For example, an observed increase in cell number may arise from the disruption of any number of biological processes and should not be interpreted as a readout for genes directly involved in the regulation of cell division. In such cases, the data is better captured by phenotype annotation.
4. Understand the data: high-throughput experiments can potentially generate large numbers of annotations and thus potentially skew subsequent analyses. It is therefore especially important that curators take time to examine all aspects of the workflow and undertake a rigorous review of the data presented. High-throughput experimental workflows are often complex and multilayered with esoteric tests and standards. Curators are encouraged to contact the authors of high-throughput papers for advice on the interpretation of the experimental findings.Within the high-throughput annotation guidelines, the GOC has provided some experiment-specific guidance. For example, for proteomics experiments, proteins should be identified by a minimum of two unique peptides [http://wiki.geneontology.org/index.php/Inferred_from_High_Throughput_Direct_Assay_(HDA)#Proteomics]. These experiment-specific guidelines will be added to by GO curators as experience in annotating high-throughput papers grows.

## Current status of high-throughput annotations in GO

Potential contributors of high-throughput annotations were identified by a search of QuickGO for papers with associated with a high number of annotations (≥65). Of 15 potential contributors identified, 13 annotation groups took part in a review of annotation sets for high-throughput data. Papers that contained ≥40 annotations to the same evidence code were selected for review—this cut-off was chosen to capture as many high-throughput papers as possible without generating unmanageably high false positive rates for reviewers. For participating annotation groups, 380 publications, representing 72 905 annotations, were identified for a review (of 417 publications; 73 028 annotations overall) and, of these, 298 have been reviewed. A total of 178 papers contained high-throughput data, but, for 39, the data did not meet the annotation standards provided in the guidelines and the corresponding annotations were deleted from GO. The annotations for 120 reviewed papers did not require re-annotation and retained their conventional evidence codes. These were usually related to papers covering in-depth characterization genes, gene families or pathways/processes with many different GO terms. Many of the participating groups have completed the revision of high-throughput paper annotations, updating evidence codes to the high-throughput equivalents. There are currently 34 533 high-throughput-evidenced annotations from 144 research publications across 23 species in the GO database. This represents 4.5% of the total number of experimentally evidenced annotations (764 600) in the GO database (data from AmiGO, 2018-12-02; 10.5281/zenodo.1899458).

## Conclusion

The GOC provides an excellent forum for annotation groups to review and harmonize curation practices. By reviewing GO annotations derived from high-throughput studies, the GOC has provided a framework to aid annotation consistency and allow GO curators to confidently annotate papers containing valuable data from high-throughput studies without the need for extra training. The major difference in how a GO curator approaches high-throughput versus low-throughput publications is the investment of time in the decision to curate—curators should examine all aspects of the workflow: experimental design, controls, data handling, validation and statistics. From this, the curator must decide whether the data is amenable to functional annotation and whether the statistical measures can be used to extract a higher confidence subset of gene products for annotation. Nevertheless, some high-throughput studies employ very complex methodology and statistics, which make the confidence level difficult to establish. In these cases, curators are advised to directly contact the authors or experts within the research community for advice. Indeed, experts in the fields of proteomics and RNAi have made valuable contributions to the high-throughput annotation guidelines. Within the GOC, discussion and documentation of challenging high-throughput papers is encouraged and as curators review and curate more high-throughput publications, they will further contribute to the GOC guidelines. This is illustrated by the work that the Functional Gene Annotation team at University College London is currently undertaking, working with leaders in the field to develop a common set of standards for the annotation of extracellular matrix components from high-throughput proteomics studies.

For consumers of GO annotation, previously unable to differentiate between annotations from hypothesis-driven gene function studies and those from high-throughput screens, the high-throughput evidence codes provide a much-needed level of transparency. Critically, the new evidence codes provide a mechanism for selecting or deselecting these annotations. The GO is frequently used in the analysis of high-throughput studies and being able to exclude data from large studies will help to avoid biases, particularly in the case where the results of similar studies have been annotated using the GO.

In summary, the provision of a set of new evidence codes and guidelines will facilitate a higher standard and consistency in the functional annotation of gene products identified in high-throughput studies and allow consumers to clearly differentiate annotations with a high-throughput provenance.

## Case studies

### Case study 1: high false-positive rate = high numbers of erroneous annotations

Publication: proteomic analysis of podocyte exosome-enriched fraction from normal human urine. PMID:23376485 (11).

Summary: proteomic analysis by liquid chromatography–tandem mass spectrometry of proteins from human urine podocyte-enriched exosome fraction using immunoadsorption to paramagnetic beads coated with a monoclonal antibody against the podocyte-specific complement receptor type 1.

GO annotations pre-review: 1714 annotations to `extracellular exosome’ (GO:0070062), direct assay evidence used in manual assertion (ECO:0000314).

GO annotations post-review: 0.

Review found: this study identifies proteins from urine by an immunoadsorption technique to `enrich’ for podocyte exosomes—it does not specifically aim to isolate and `purify’ podocyte exosomes. It is not suitable for annotation as the experimental workflow results in a significant number of non-exosomal protein contaminations, which would be impossible to differentiate without additional studies. This paper has been marked as `Not suitable for annotation’ in the Protein2GO annotation tool (12).

### Case study 2: misinterpretation of experiment = high numbers of erroneous annotations

Publication: genome-wide analysis of self-renewal in *Drosophila* neural stem cells by transgenic RNAi. PMID:21549331 (13).

Summary: genome-wide transgenic RNAi to identify genes potentially involved in controlling the balance between stem cell self-renewal and differentiation in *Drosophila melanogaster* neuroblasts. Identify genes causing visible defects in neuroblast lineages and quantify phenotypes by measurements of proliferation, lineage, cell size and cell shape.

GO annotations pre-review: 549 annotations to neurogenesis (GO:0022008), mutant phenotype evidence used in manual assertion (ECO:0000315).

GO annotations post-review: 0.

Review found: The authors were interested in `how the balance between self-renewal and differentiation is controlled within a stem cell lineage’ and look for cell division, growth and proliferation defects. This study is not sufficiently targeted to allow the confident assignment of GO terms to genes. The phenotypes may arise from the disruption of any number of processes. No attempt is made to specifically relate this to neurogenesis alone and many of these genes are so called `housekeeping’ genes. This paper has been marked as `Not suitable for annotation’ in the Protein2GO annotation tool (12).

This data was better represented by FlyBase phenotype curation. A total of 608 alleles were curated, resulting in 2196 phenotype annotations.

### Case study 3: use of multivariate data analysis to reduce false positives in organelle isolation

Publication: Mapping organelle proteins and protein complexes in *D. melanogaster*. PMID:19317464 (14).

Summary: Organelles were partially purified from *Drosophila* embryos by density gradient centrifugation. Trypsin-digested proteins from gradient fractions were labeled with iTRAQ and analyzed using LC–MS/MS.

Annotations pre-review: 329 annotations to either plasma membrane (GO:0005886), endomembrane system (GO:0012505) or mitochondrion (GO:0005739), direct assay evidence used in manual assertion (ECO:0000314).

Annotations post-review: 318 annotations to either plasma membrane (GO:0005886), endomembrane system (GO:0012505) or mitochondrion (GO:0005739), high-throughput direct assay evidence used in manual assertion (ECO:0007005); 7 annotations (direct assay evidence used in manual assertion, ECO:0000314).

Review found: The use of multivariate data analysis on what is a relatively crude preparation reduces the issues from contaminants, allowing relatively high confidence in assigning correct subcellular locations. A total of 11 annotations were removed, as the proteins were only identified by a single unique peptide. The remaining 318 annotations were updated to a high-throughput evidence code. From fluorescence microscopy studies of individual proteins, seven annotations were also made using the conventional evidence code. Protein complexes were not annotated from this study as the authors did not specifically set the experiment up to address this question, and predicted localization was used for classification.

## Notes

**Database URL:** http://geneontology.org/
